# Frailty among inpatients with Schizophrenia: Status, influencing factors, and their correlation with quality of life

**DOI:** 10.3389/fpsyt.2022.1067260

**Published:** 2023-01-04

**Authors:** Cui Yang, Xiaofeng Hou, Xiucheng Ma, Dongmei Wu

**Affiliations:** ^1^School of Nursing, Chengdu Medical College, Chengdu, China; ^2^Department of Nursing, The Clinical Hospital of Chengdu Brain Science Institute, MOE Key Laboratory for Neuroinformation, University of Electronic Science and Technology of China, Chengdu, China

**Keywords:** frailty, influencing factors, quality of life, correlation research, Schizophrenia

## Abstract

**Objective:**

This study aimed to understand frailty and its influencing factors in inpatients with Schizophrenia in Chengdu and to explore correlations between frailty and quality of life.

**Methods:**

From May to July 2022, inpatients with Schizophrenia were surveyed using a general information questionnaire, frailty phenotype (FP) scoring, the Self-Rating Depression Scale (SDS), the Mini-Mental State Examination (MMSE), and the SZ Quality of Life Scale (SQLS). Multivariate logistic regression was conducted to assess factors influencing frailty and multivariate linear regression was conducted to assess the factors influencing quality of life.

**Results:**

A total of 556 hospitalized patients with Schizophrenia were included and divided into three groups according to the degree of frailty, of which 153 cases (27.5%) were without frailty, 348 cases (62.6%) were in early frailty, and 55 cases (9.9%) were in frailty. Univariate analysis of age, history of falls during hospitalization, polypharmacy, compulsory treatment during hospitalization, self-reported health status, activity level, cognitive impairment, depressive symptoms, “psychology and society,” “motivation and energy” and “symptoms and side-effects” showed statistically significant differences between the groups. Multinomial logistic regression showed that age, BMI, self-reported health, activity, cognitive impairment, motivation and energy, and symptoms and side-effects were influencing factors for frailty in hospitalized patients with Schizophrenia. Correlation analysis shows that frailty score positively correlated with SQLS score.

**Conclusion:**

We found that frailty was prevalent and that frailty was positively correlated with SQLS scores in inpatients with Schizophrenia. To effectively manage the frailty of hospitalized patients with Schizophrenia, medical staff should pay attention to its influencing factors and quality of life.

## Background

Schizophrenia (SZ) is a severe mental disorder of unknown etiology often with onset in early adulthood, recurrent relapses, a high disability rate, and a significant increase in the economic burden on families and society (1). Due to the chronic and reoccurring nature of the disease, the heavy burden of family care, and commonly insufficient community medical care, patients with SZ often require long-term hospitalization (2).

*Frailty* is defined as a decrease in the body’s multisystem reserve capacity that increases an individual’s susceptibility to small internal and external stimuli (3, 4). Frailty is usually assessed in older adults, and identifying frailty in adults under 60 may also be of value (5, 6). Some vulnerable groups with chronic diseases may be at risk of debilitation early in life (7–12). The accelerated aging hypothesis of SZ (13–17) states that physiological changes in patients with SZ tend to occur earlier than in the general population. Basic research (18–20) has shown that leukocyte telomere length, a new marker of cellular aging, is significantly shortened in patients with SZ, suggesting that they are more likely to age prematurely than the general population.

Quality of life is divided into the objective quality of life and subjective life satisfaction (20). Due to changes in medical models, the treatment goals and prognosis evaluation for SZ are no longer limited to the relief of clinical symptoms but also include comprehensive improvement of quality of life (21). Subjective quality of life in SZ is a subjective index for evaluating treatment effects, which can effectively reflect the current health status of the human body and the satisfaction with physical, psychological, social, and behavioral functions. This can guide policies and practices for patients with mental disorders (22).

Frailty increases the risk of adverse health outcomes (23), affects the employment outcomes of patients with SZ (24), increases the physical and psychological burden, and reduces the social quality of life. The relationship between frailty and quality of life is bidirectional (25), with poor quality of life leading to increased risk of frailty and frailty predisposing people to low quality of life, so understanding the relationship between frailty and quality of life is critical. Although studies in older age groups and patients with cancer have demonstrated a link between frailty and quality of life (26, 27), an extensive literature search found few studies on frailty in hospitalized patients with SZ and no evidence on the relationship between frailty and quality of life. Thus, the present study intended to explore the status of and factors contributing to frailty in inpatients with SZ and the correlation between frailty and quality of life.

## Materials and methods

### Sample characteristics

From May to July 2022, we facilitated a sample of inpatients with SZ from four psychiatric hospitals in Chengdu. Inclusion criteria: hospitalized patients aged ≥ 18 years; diagnosis by attending psychiatrists meeting the diagnostic criteria for SZ in the 10th edition of the International Classification of Diseases (ICD-10) (28); Most of the treated psychiatric symptoms disappeared after hospitalization and stabilized during the investigation period, with a score of < 60 on the Positive and Negative Symptom Scale (PANSS) (29); No visual, hearing, or communication impairments (or these could be corrected). Exclusion criteria: comorbid intellectual disability, dementia, or other mental disorders; severe physical disease or brain organic disease; poor compliance, severe aggressive behavior, or suicidality. The Fourth People’s Hospital of Chengdu Ethics Committee approved this study.

There following five questionnaires were included: demographic data, the Fried frailty phenotype (FP) (3), the Mini-Mental State Examination (MMSE) (30), the Self-Rating Depression Scale (SDS) (31) and the SZ Quality of Life Scale (SQLS) (32). Researchers screened the participants who met the inclusion and exclusion criteria and distributed questionnaires, using unified guidelines to explain the research purpose and significance. The participants filled out the questionnaires independently, and the researchers collected them immediately and checked each item to ensure completeness. If the participants did not understand the content of any items, the researchers provided explanations according to the respective scale guide.

### Outcomes

The FP (3) was used to screen the frailty of hospitalized patients with SZ. It includes five items: unexpected weight loss, fatigue, loss of grip strength, slowed pace, and reduced fitness. *Frailty syndrome* is defined as the presence of three or more items, fewer than three items is categorized as pre-frailty, and zero items means no frailty. The FP focuses on physical weakness of the body using objective and quantitative evaluation indicators and has a high predictive ability for weakened states. Tsai et al. (33) also used this to evaluate frailty in hospitalized patients with SZ in Taiwan. The present study measured grip strength using a Camry digital grip dynamometer (EH101; South El Monte, CA, USA). Patients were instructed to stand upright, with both feet separated and arms hanging naturally. Full-force grip strength was measured in one hand, and the maximum value of three measurements was recorded. Moreover, pace was measured with patients instructed to walk naturally for 4.5 m from a starting point, and the time was recorded with a stopwatch; the minimum value over three measurements was used.

The MMSE, developed by Folstein et al. (30) in 1975 and one of the most influential cognitive function screening tools, was used to assess the cognitive status of hospitalized patients with SZ. The MMSE consists of 30 items covering orientation, memory, attention and calculation, recall ability, and language ability. The scale is easy to operate and suitable for evaluating cognitive dysfunction in SZ (34). The scale has a maximum score of 30 points, with higher scores indicating better cognitive function; ≥ 27 points indicates normal cognitive function and < 27 indicates cognitive dysfunction.

The SDS, created by Zung et al. (31) was used to assess depressive symptoms. The scale consists of 20 items and reflects subjective feelings of patients by evaluating the frequency of depressive symptoms corresponding to each item. A 4-level rating was used: 1 point, for no or very low frequency, 2 points for low frequency, 3 points for a considerable frequency, and 4 points for high or continuous frequency; Ten items were scored in reverse. The total score of each item was the raw score, and this was multiplied by 1.25 and rounded up to the standard score. The SDS standard score ranges from 25 to 100 points. The higher the score, the more severe the depressive state, with good reliability and validity (35). Patients with an SDS standard score ≥ 50 were considered to have depressive symptoms.

The SQLS was used to evaluate inpatient quality of life. The scale was developed by British psychiatrist Greg Wilkingsony (32) in 1999 and includes three scales with a total of 30 items: psychosocial, motivation and energy, and symptoms and side-effects, using a 5-point scale (0 = “never”, 1 = “occasionally”, 2 = “sometimes”, 3 = “often”, 4 = “always”). Items 12, 13, 15 and 20 are reverse-scored. Each subscale is calculated as follows: psychosocial subscale = rough score of 15 items/(4 × 15) × 100; energy/motivation subscale = rough score of 7 items/(4 × 7) × 100; symptom/adverse response subscale = crude score of 8 items/(4 × 8) × 100. The total score for each subscale is 0 to 100, and the higher the score, the worse the subjective quality of life. Luo (36) introduced a translated Chinese version of the SQLS in 2002. The test-retest reliability of the Chinese version is 0.87, and Cronbach’s α is 0.70–0.92. It has good reliability and validity and is suitable for assessment of the quality of life in patients with SZ.

### Covariates

The socioeconomic and demographic factors selected were age group (18–44 years, 45–59 years, and 60 years or older); sex (male, female), education (primary school and below, junior school, high school, college and above); family monthly income (1000 Chinese yuan and less, 1,000–3,000 yuan, 3,001–5,000 yuan, 5,000 yuan and more); and frequency of family visits (none, 1 to 2 times/month, 3 times or more/month).

Factors used to assess patient health were age of onset (18 years below, 18–44 years, 45–59 years, and 60 years and older); disease duration (5 years below, 5–10 years, and10 years and above); smoking history (yes/no); history of falls during hospitalization (yes/no); family history of psychosis (yes/no); polypharmacy (yes/no); compulsory treatment during hospitalization (yes/no); activity levels (normal/low/inactive); self-reported health (good/fair/poor); and body mass index (BMI; calculated based on height and weight).

### Statistical analysis

SPSS statistical software (v. 26.0; IBM Corp., Armonk, NY, USA) was used for data analysis. Count data are expressed as frequency and percentage, measurement data obeyed normal distribution and are expressed as the means and standard deviations. The Chi-squared test, Student’s t test and Analysis of Variance were used to compare groups. Logistic regression was used to analyze the influencing factors of frailty and Linear regression analysis was used to analyze the influencing factors of quality of life. Non-frail or frail was the dependent variable, and considering the interference factors, the univariate analysis of demographic data, age, sex, education, family monthly income, frequency of family visits, age of onset, smoking history, history of falls during hospitalization, family history of psychosis, polypharmacy, compulsory treatment during hospitalization, self-reported health, activity level, cognitive impairment, depressive symptoms, disease duration and SQLS scores with all variables were independent variables except for the dependent variable. SQLS scores was the dependent variable, and the inclusion method of independent variables was the same as that of frailty influencing factor analysis. The correlation between frailty and SQLS scores was analyzed. Spearman correlation analysis was used because the data did not follow a normal distribution. A p value < 0.05 was considered statistically significant.

## Results

### Participants

A total of 570 hospitalized patients with SZ met the inclusion and exclusion criteria, and the number of completed questionnaires was 556 (97.5%). The sample had the following characteristics: most of the participants were male, patients were between 45 and 59 years old, and 31.1% had finished high school or technical secondary school education; 45.0% had a BMI of 18.5–23.9 kg/m^2^; 35.3% has a monthly household income 1,000–3,000 Chinese yuan; 44.6% had a frequency of family visits of 0 times/month; 85.3% had an age at first SZ diagnosis of 18–44 years old; 48.0% had a history of smoking; 22.8% had a history of falls during hospitalization; 18.9% had a family history of mental illness; 41.4% had polypharmacy; and 41.9% received compulsory treatment during hospitalization. The average SQLS score was 83.82 ± 40.30.

### Participant demographic characteristics and frailty

The characteristics of the participants based on different degrees of frailty are shown in Table 1. There were 153 cases (27.5%) of no frailty, 348 (62.6%) of pre-frailty, and 55 (9.9%) of frailty, and the mean frailty score of the 556 participants was 1.17 ± 1.00. As can be seen in Table 1, age, history of falls during hospitalization, polypharmacy, compulsory treatment during hospitalization, self-reported health status, activity level, cognitive impairment, depressive symptoms, “psychology and society,” “motivation and energy,” and “symptoms and side-effects” showed statistically significant differences among patients with different degrees of frailty (*P* < 0.05). The occurrence of frailty is shown in Table 2.

**TABLE 1:** Characteristics of the participants based on different frailty (*n* = 556).

Variables	*n* (%)	Non-frail (*n* = 153)	Pre-frail (*n* = 348)	Frail (*n* = 55)	F/x^2^	*P*
Age^a^					13.378	< 0.001
18–44	173 (31.1)	70 (45.7)	95 (27.3)	8 (14.5)		
45–59	284 (51.1)	64 (41.8)	191 (54.9)	29 (52.7)		
≥ 60	99 (17.8)	19 (12.5)	62 (17.8)	18 (32.7)		
Sex					4.941	0.085
Male	350 (62.9)	86 (56.2)	231 (66.4)	33 (60.0)		
Female	206 (37.1)	67 (43.8)	117 (33.6)	22 (40.0)		
Education					3.987	0.019
Primary school and below	104 (18.7)	30 (19.6)	56 (16.1)	18 (32.7)		
Junior school	155 (27.9)	42 (27.5)	97 (27.9)	16 (29.1)		
High school	173 (31.1)	41 (26.8)	119 (34.2)	13 (23.6)		
College and above	124 (22.3)	40 (26.1)	76 (21.8)	8 (14.5)		
BMI [kg/m^2^]					0.090	0.914
<18.5	30 (5.4)	7 (4.6)	18 (5.2)	5 (9.1)		
18.5–23.9	250 (45.0)	71 (46.4)	156 (44.8)	23 (41.8)		
24–27.9	161 (29.0)	46 (30.1)	100 (28.7)	15 (27.3)		
≥28	115 (20.7)	29 (19.0)	74 (21.3)	12 (21.8)		
Family monthly income [Chinese yuan]					3.230	0.040
<1,000	158 (28.4)	34 (22.2)	104 (29.9)	20 (36.4)		
1,000–3,000	196 (35.3)	55 (35.9)	123 (35.3)	18 (32.7)		
3,001–5,000	115 (20.7)	33 (21.6)	70 (20.1)	12 (21.8)		
>5,000	87 (15.6)	31 (20.2)	51 (14.7)	5 (9.1)		
Frequency of family visits [times/month]					2.568	0.078
0	248 (44.6)	59 (38.6)	157 (45.1)	32 (58.2)		
1-2	237 (42.6)	71 (46.4)	149 (42.8)	17 (30.9)		
≥ 3	71 (12.8)	23 (15.0)	42 (12.1)	6 (10.9)		
Age of onset [years]					1.091	0.337
<18	43 (7.7)	12 (7.8)	26 (7.5)	5 (9.1)		
18–44	474 (85.3)	131 (85.6)	297 (85.3)	46 (83.6)		
45–59	35 (6.3)	9 (5.9)	23 (6.6)	3 (5.5)		
≥60	4 (0.7)	1 (0.7)	2 (0.6)	1 (1.8)		
Smoking history					0.020	0.990
Yes	267 (48.0)	74 (48.4)	167 (48.0)	26 (47.3)		
No	289 (52.0)	79 (51.6)	181 (52.0)	29 (52.7%)		
History of falls during hospitalization					28.721	<0.001
Yes	127 (22.8)	21 (13.7)	79 (22.7)	27 (49.1)		
No	429 (77.2)	132 (86.3)	269 (77.3)	28 (50.9)		
Family history of psychosis					1.642	0.440
Yes	105 (18.9)	33 (21.6)	60 (17.2)	12 (21.8)		
No	451 (81.1)	120 (78.4)	288 (82.8)	43 (78.2)		
Polypharmacy					9.197	0.010
Yes	230 (41.4)	49 (32.0)	152 (43.7)	29 (52.7)		
No	326 (58.6)	104 (68.0)	196 (56.3)	26 (47.3)		
Compulsory treatment during hospitalization					8.687	0.013
Yes	233 (41.9)	73 (47.7)	130 (37.4)	30 (54.5)		
No	323 (58.1)	80 (52.3)	218 (62.6)	25 (45.5)		
Self-reported health^a^					41.517	<0.001
Good	378 (68.0)	126 (82.4)	237 (68.1)	15 (27.3)		
Fair	132 (23.7)	26 (17.0)	83 (23.9)	23 (41.8)		
Poor	46 (8.3)	1 (0.7)	28 (8.0)	17 (30.9)		
Activity level^a^					71.448	<0.001
Normal	427 (76.8)	142 (92.8)	273 (78.4)	12 (21.8)		
Low	107 (19.2)	8 (5.2)	66 (19.0)	33 (60.0)		
Inactive	22 (4.0)	3 (2.0)	9 (2.6)	10 (18.2)		
Cognitive impairment					28.855	<0.001
Yes	249 (44.8)	44 (28.8)	168 (48.3)	37 (67.3)		
No	307 (55.2)	109 (71.2)	180 (51.7)	18 (32.7)		
Depressive symptoms					47.390	<0.001
Yes	197 (35.4)	35 (22.9)	121 (34.8)	41 (74.5)		
No	359 (64.6)	118 (77.1)	227 (65.2)	14 (25.5)		
Disease duration [years]					4.834	0.008
< 5	43 (7.7)	17 (11.1)	25 (7.2)	1 (1.8)		
5-10	90 (16.2)	32 (20.9)	51 (14.7)	7 (12.7)		
> 10	423 (76.1)	104 (68.0)	272 (78.2)	47 (85.5)		
SQLS [X **± *S*]**						
Psychology and society^a^	26.08 ± 18.26	17.37 ± 12.41	27.27 ± 17.49	42.73 ± 22.80	47.935	<0.001
Motivation and energy^a^	37.93 ± 14.97	31.09 ± 13.91	38.29 ± 13.73	54.68 ± 11.35	61.437	<0.001
Symptoms and side-effects^a^	19.81 ± 16.06	11.15 ± 9.29	20.85 ± 15.45	37.33 ± 18.43	69.467	<0.001

^a^*Post hoc* test *P* < 0.05.

**TABLE 2:** The incidence of frailty in hospitalized patients with Schizophrenia (*n* = 556).

Frailty indicator	Yes	No
**Weight loss:** Any unexpected weight loss > 4.5kg or > 5% in the past year	58	498
**Fatigue** Did the following happen to you frequently in the past week? Choose a question to answer: (1) It feels laborious to do anything. (2) I feel unable to continue to perform my daily work.	124	432
**Grip strength drops** (BMI, kg/m^2^; grip strength, kg) Male: BMI ≤ 24, grip strength ≤ 29; BMI 24.1–26, grip strength ≤ 30; BMI 26.1–28, grip strength ≤ 30; BMI ≥ 28, grip strength ≤ 32 Female: BMI ≤ 23, grip strength ≤ 17; BMI ≤ 23.1–26, grip strength ≤ 17.3; BMI ≤ 26.1–29, grip strength ≤ 18; BMI ≥ 29, grip strength ≤ 21	41	515
**Slow pace** Male ≤ 173 cm, ≥ 7 s; > 173 cm, ≥ 6 s Female ≤ 159 cm, ≥ 7 s; > 159 cm, ≥ 6 s	306	250
**Physical decline:** Male: < 383 kcal/week, female: < 270 kcal/week	121	435

### Participant demographic characteristics and SQLS

The characteristics of the participants based on SQLS are shown in Table 3. The mean SQLS score of the 556 participants was 83.82 ± 40.30. There were statistically significant differences among inpatients with SZ across the following variables: history of falls during hospitalization, family history of psychosis, polypharmacy, compulsory treatment during hospitalization, self-reported health, Cognitive impairment, Depressive symptoms, and frailty (*P* < 0.05).

**TABLE 3:** Demographic characteristics of the participants and SQLS (*n* = 556).

Variables	SQLS score	Psychology and society	Motivation and energy	Symptoms and side-effects
**Age**				
18–44	81.554 ± 38.823	27.524 ± 18.272	37.448 ± 14.520	16.582 ± 14.329
45–59	87.098 ± 40.676	26.895 ± 18.186	38.317 ± 14.647	21.886 ± 16.771
≥ 60	78.365 ± 41.269	21.195 ± 17.795	37.662 ± 16.706	19.507 ± 16.009
F	2.129	4.422*	0.199	5.993**
**Sex**				
Male	83.320 ± 38.821	25.780 ± 17.455	37.816 ± 14.875	19.723 ± 15.510
Female	84.665 ± 42.774	26.57 ± 19.575	38.124 ± 15.171	19.963 ± 16.980
T	0.380	0.497	0.234	0.170
**Education**				
Primary school and below	88.959 ± 42.799	27.339 ± 18.225	38.873 ± 16.059	22.746 ± 16.607
Junior school	83.197 ± 40.111	25.752 ± 18.892	38.110 ± 15.043	19.334 ± 15.761
High school	85.113 ± 40.332	26.368 ± 18.326	38.748 ± 14.665	19.996 ± 16.177
College and above	78.476 ± 38.086	25.013 ± 17.505	35.771 ± 14.321	17.691 ± 15.591
F	1.365	0.336	1.178	1.942
**BMI**				
<18.5	82.757 ± 37.316	23.055 ± 19.093	39.285 ± 12.192	20.870 ± 15.867
18.5–23.9	82.427 ± 41.363	25.046 ± 18.066	37.942 ± 15.853	19.334 ± 16.731
24–27.9	85.686 ± 39.610	27.101 ± 18.431	38.087 ± 14.026	20.488 ± 15.952
≥ 28	84.506 ± 40.036	27.666 ± 18.205	37.329 ± 15.087	19.510 ± 15.036
F	0.231	0.998	0.149	0.169
**Family monthly income (Chinese yuan)**				
<1,000	93.161 ± 42.118	29.124 ± 18.734	40.302 ± 15.216	23.734 ± 17.141
1,000∼3,000	82.098 ± 38.045	25.773 ± 17.399	37.718 ± 14.490	18.606 ± 15.195
3,001∼5,000	82.213 ± 42.556	24.884 ± 19.559	37.546 ± 15.490	19.782 ± 16.225
>5,000	72.848 ± 35.591	22.796 ± 16.925	34.605 ± 14.423	15.445 ± 14.275
F	5.279**	2.607	2.817*	5.803**
**Frequency of family visits (times/month)**				
0	87.757 ± 41.866	27.090 ± 18.062	39.372 ± 15.568	21.295 ± 16.563
1-2	80.036 ± 36.759	24.507 ± 17.401	36.844 ± 14.016	18.684 ± 14.830
≥ 3	82.686 ± 45.061	27.769 ± 21.341	36.519 ± 15.698	18.397 ± 17.893
F	2.336	1.566	2.097	1.887
**Age of onset (years)**				
<18	87.699 ± 34.495	28.023 ± 18.028	41.362 ± 15.316	18.313 ± 13.285
18–44	83.292 ± 40.837	25.956 ± 18.482	37.590 ± 14.742	19.745 ± 16.322
45–59	83.898 ± 38.959	25.238 ± 15.801	36.428 ± 15.920	22.232 ± 15.966
≥ 60	103.78 ± 53.245	26.666 ± 18.807	54.464 ± 21.699	22.656 ± 14.518
F	0.486	0.195	2.600	0.433
**Smoking history**				
Yes	83.209 ± 41.406	26.248 ± 18.541	37.051 ± 15.197	19.908 ± 16.750
No	84.382 ± 39.310	25.916 ± 18.019	38.741 ± 14.742	19.723 ± 15.415
T	0.343	−0.214	1.331	−0.136
**History of falls during hospitalization**				
Yes	100.718 ± 44.440	31.286 ± 20.454	41.676 ± 15.179	17.455 ± 1.548
No	78.815 ± 37.611	24.533 ± 17.279	36.821 ± 14.747	14.843 ± 0.716
T	−5.521**	−3.380**	−3.236**	−6.585**
**Family history of psychosis**				
Yes	92.69 ± 48.23	30.111 ± 21.111	37.993 ± 15.481	24.583 ± 18.108
No	81.75 ± 37.98	25.136 ± 17.417	37.915 ± 14.869	18.701 ± 15.349
T	−2.171*	−2.243*	−0.048	−3.413**
**Polypharmacy**				
Yes	88.262 ± 40.165	28.043 ± 18.582	38.928 ± 13.859	21.290 ± 17.327
No	80.683 ± 40.158	24.688 ± 17.921	37.226 ± 15.693	18.769 ± 15.034
T	−2.191*	−2.141*	−1.349	−1.783
**Types of antipsychotic drugs**				
New	85.896 ± 40.514	27.091 ± 18.562	38.353 ± 15.142	20.451 ± 16.263
Traditional	81.757 ± 40.419	25.057 ± 17.995	37.546 ± 14.820	19.154 ± 15.961
Both	66.404 ± 23.011	17.833 ± 10.888	33.571 ± 13.489	15.000 ± 10.395
F	1.658	1.872	0.624	0.893
**Compulsory treatment during hospitalization**				
Yes	89.593 ± 40.729	28.497 ± 18.308	39.193 ± 14.877	21.901 ± 16.591
No	79.653 ± 39.525	24.329 ± 18.045	37.019 ± 14.998	18.304 ± 15.509
T	−2.888**	−2.671**	−1.692	−2.620**
**Self-reported health^a^**				
Good	74.196 ± 35.248	22.477 ± 16.439	35.638 ± 14.585	16.079 ± 14.162
Fair	95.511 ± 40.023	29.974 ± 18.791	40.395 ± 14.108	25.142 ± 16.589
Poor	129.336 ± 40.743	44.456 ± 17.881	49.689 ± 14.142	35.190 ± 15.994
F	54.478**	38.176**	21.941**	44.656**
**Activity level**				
Normal	115.497 ± 44.297	35.303 ± 19.898	50.649 ± 15.919	29.545 ± 17.723
Low	103.212 ± 45.610	33.769 ± 21.508	45.026 ± 14.894	24.415 ± 18.503
Inactive	77.326 ± 36.233	23.672 ± 16.569	35.496 ± 14.051	18.157 ± 14.906
F	21.134**	12.966**	28.093**	11.099**
**Cognitive impairment**				
Yes	87.925 ± 41.232	26.653 ± 18.415	39.371 ± 15.672	21.900 ± 16.493
No	80.488 ± 39.277	25.608 ± 18.142	36.761 ± 14.299	18.118 ± 15.513
T	−2.171*	−0.671	−2.050*	−2.778**
**Depressive symptoms**				
Yes	114.020 ± 39.142	38.426 ± 18.659	47.008 ± 12.833	28.585 ± 17.483
No	67.245 ± 30.024	19.298 ± 14.003	32.948 ± 13.680	14.998 ± 12.911
T	−14.582**	−12.575**	−11.845**	−9.569**
**Disease duration**				
<5	82.248 ± 38.161	27.015 ± 19.805	37.790 ± 14.791	17.441 ± 13.522
5-10	80.143 ± 41.899	26.388 ± 17.716	36.706 ± 15.728	17.048 ± 16.289
>10	84.760 ± 40.206	25.914 ± 18.247	38.204 ± 14.849	20.641 ± 16.185
F	0.521	0.087	0.373	2.377
**Degree of frailty^a^**				
Non-frail	59.619 ± 24.172	17.374 ± 12.405	31.092 ± 13.910	11.151 ± 9.290
Pre-frail	86.411 ± 37.336	27.270 ± 17.493	38.290 ± 13.732	20.851 ± 15.454
Frail	134.732 ± 41.780	42.727 ± 22.798	54.675 ± 11.353	37.329 ± 18.433
F	97.244**	47.935**	77.138**	69.467**

^a^*Post hoc* test *P* < 0.05; *P < 0.05; **P < 0.01.

### Logistic regression analysis of factors related to frailty

The factors influencing frailty were analyzed. Different degrees of frailty were the dependent variable and remaining variables were used as independent variables. Multiple logistic regression analysis revealed that age, BMI, self-rated health status, activity level, cognitive impairment, “motivation and energy,” and “symptoms and side-effects” were associated with frailty. The results are shown in Table 4.

**TABLE 4:** Logistic regression analysis of factors related to frailty.

Variables	*B*	*SE*	*Waldχ^2^*	*DF*	*P*	*OR*	*95% CI*
Age = 18–44	-2.623	0.961	7.445	1	0.006	0.073	[0.011,0.478]
BMI = 18.5–23.9	-1.729	0.704	6.028	1	0.014	0.177	[0.045,0.706]
Self-reported health = Good	-3.031	1.253	5.854	1	0.016	0.048	[0.004,0.562]
Activity level = Inactive	2.493	1.066	5.470	1	0.019	12.102	[1.498,97.787]
Activity level = Low	3.675	0.700	27.531	1	<0.001	39.464	[9.999,155.757]
Cognitive impairment = Yes	-1.403	0.571	6.039	1	0.014	0.246	[0.080,0.753]
Motivation and energy	0.121	0.025	22.994	1	<0.001	1.128	[1.074,1.186]
Symptoms and side-effects	0.080	0.022	12.975	1	<0.001	1.084	[1.037,1.132]

All the study variables were entered into a logistic regression model. Abbreviations: SE, Standard error. OR, Odds ratio. CI, confidence interval.

### Linear regression analysis of factors related to quality of life

The influencing factors of quality of life were analyzed. The SQLS score was the dependent variable, and remaining variables were used as independent variables. Multiple linear regression analysis showed that age, sex, family monthly income, falls history during hospitalization, self-reported health, activity level, depressive symptoms, frailty were the main influencing factors related to quality of life for patients with SZ. The results are shown in Table 5.

**TABLE 5:** Multivariable linear regression analysis of factors related to quality of life.

Variable	*B*	*SE*	β	*t*	*P*	*95% CI*	*VIF*	*Adjusted R2*	*F*
Final model								0.490	30.672
Age	-5.455	2.315	-0.093	-2.357	0.019	[−10.002,−0.908]	1.697		
Sex	-7.203	3.172	-0.086	-2.271	0.024	[−13.434,−0.972]	1.576		
Family monthly income	-3.573	1.358	-0.091	-2.631	0.009	[−6.241,−0.905]	1.315		
Falls history during hospitalization	7.648	3.093	0.080	2.473	0.014	[1.572,13.724]	1.133		
Family history of psychosis	8.210	3.211	0.080	2.557	0.011	[1.902,14.518]	1.061		
Self-reported health	9.709	2.148	0.154	4.519	<0.001	[5.489,13.929]	1.259		
Activity level	-5.767	2.618	-0.075	-2.203	0.028	[−10.910,−0.624]	1.276		
Depressive symptoms	32.820	2.827	0.390	11.609	<0.001	[27.267,38.374]	1.229		
Frailty	21.339	2.473	0.310	8.629	<0.001	[16.481,26.198]	1.410		

All the study variables were entered into a multivariable linear regression model. VIF, the variance inflation factor.

### Analysis of the correlation between frailty and quality of life

The frailty and SQLS scores did not follow a normal distribution, and Spearman’ s test was used to analyze the correlation between frailty and quality of life. The results of the correlation analysis showed that frailty was positively correlated with quality of life (*R* = 0.511, *P* < 0.001). The higher the frailty score is, the worse the quality of life.

## Discussion

The present study showed a frailty rate slightly lower than that of a study in Taiwan (10.2%) using the same criteria (33). Differences in frailty rates may be related to economic conditions and medical service levels. The survey areas of the present study are in new first-tier cities with relatively developed economies and suitable mental health treatment centers. These cities can provide vocational rehabilitation training with good treatment effects, such as agricultural therapy, garden therapy, and manual training.

Our study showed that age, BMI, motivation and energy, symptoms and side-effects, self-rated health status, activity level and cognitive impairment were the influencing factors related to frailty in inpatients with SZ. Age and BMI have been shown to be a risk factor for frailty (37, 38). “Motivation and energy” and “symptoms and side-effects” were associated with frailty in the present study, indicating that insufficient motivation and energy and severe symptoms and side-effects would increase the risk of frailty. The reason may be that the strong sedative effect causes a lack of subjective motivation and energy, which leads to laziness, limited activity, and frailty (39). Self-reported health is a widely used health criterion to predict and identify frailty (40, 41). The concept of frailty self-perception can explain the relationship between self-rated health status and frailty. Patients with a negative self-perception of frailty are more likely to develop frailty, and a positive self-perception of frailty can delay the development of frailty, and expressive symptoms and cognitive status can mediate the relationship between frailty self-perception and frailty (42). A large cross-sectional study in China (43) showed that poor self-rated health status was a risk factor for frailty in the elderly, similar to the results of the present study. A sedentary lifestyle is a significant risk factor for the progression and morbidity of frailty (44, 45). A study (46) with a large sample showed that the duration of sedentary periods could help judge the existence of frailty and predict the development of frailty. The present study showed that patients with low or moderate activity levels were more prone to frailty than those with higher activity levels, and this is similar to the study (47). Therefore, medical staff should observe the activity of hospitalized patients with SZ, help them formulate activity plans, and urge them to exercise to maintain a healthy lifestyle. Cognitive impairment may be an early marker of physical weakness (48). A cross-sectional study (49) showed that the incidence of frailty was higher in patients with dementia, and the risk of dementia in patients with frailty was also significantly higher. People with cognitive impairment and frailty had a six-fold higher risk of death and a 13-fold higher risk of functional disability than those with only frailty or cognitive impairment. The present study showed that cognitive impairment was a risk factor for frailty, and the risk of frailty in patients with cognitive impairment was significantly higher than their counterparts. This may be because cognitive impairment and frailty share common physiological mechanisms, such as genetic inheritance, chronic inflammation, malnutrition, mitochondrial dysfunction, oxidative stress, hypothalamic–pituitary–adrenal axis dysfunction, endocrine disorders, and energy metabolism imbalances (50). Therefore, it is of great significance to explore the relationship more deeply between cognitive impairment and frailty in patients with SZ and whether reducing or delaying frailty can improve cognition.

We also found that frailty were the influencing factor related to quality of life for patients with SZ. Previous studies have shown that frailty may lead to adverse health outcomes such as falls, fractures, and hospital admissions, increasing disease burden and reducing quality of life (33). Disability-adjusted life years (DALY) is one of the essential measures of disease burden. A large European study (51) showed that frailty was significantly associated with DALY, with frail individuals having significantly higher mean DALYs than non-frail individuals. Therefore, frailty may be related to quality of life. The relationship between frailty and quality of life has long been demonstrated in older populations (52, 53). The frailty phenotypes in the elderly are inversely related to the quality of life (54). The present study showed that frailty score was positively correlated with the SQLS score, indicating that the worse the frailty, the worse the quality of life. Furthermore, poor quality of life may increase frailty in turn (55). Therefore, we speculated that the relationship between frailty and quality of life may be reciprocal. Other studies have not reported a significant cross-sectional relationship between frailty and quality of life in hospitalized patients with SZ, so this relationship requires further research.

## Limitations

The present study has several limitations. First, this was a cross-sectional survey. Although it suggested the suspected risk factors for frailty, it did not consider the temporal and causal relationships between exposures and outcomes. We also could not track the health status in all patients, especially those with poor health statuses, such as those with cognitive impairment, depressive symptoms, a history of falls during hospitalization, polypharmacy, compulsory treatment during hospitalization, and self-reported poor health. These factors require further longitudinal studies. Second, the participants were not randomly selected, so the results cannot be generalized to all patients with SZ in psychiatric hospitals. Finally, we did not notice correlation between PANSS score and grade of frialty and daily equivalent dosage of antipsychotics was not examined.

## Conclusion

We found that frailty was prevalent and the influencing factors were complex. Frailty syndrome was positively associated with quality of life in hospitalized patients with SZ. Psychiatric medical staff should pay attention to the quality of life of patients with frailty, screen for early identification of patients with pre-frailty, and formulate targeted prevention and intervention measures to improve quality of life further and delay the onset of frailty.

## Data availability statement

The raw data supporting the conclusions of this article will be made available by the authors, without undue reservation.

## Ethics statement

The studies involving human participants were reviewed and approved by The Fourth People’s Hospital of Chengdu Ethics Committee. The patients/participants provided their written informed consent to participate in this study.

## Author contributions

All authors contributed to conceiving, researching, and writing of this manuscript and approved the submitted version.
